# Dietary and Protective Factors to Halt or Mitigate Progression of Autoimmunity, COVID-19 and Its Associated Metabolic Diseases

**DOI:** 10.3390/ijms22063134

**Published:** 2021-03-19

**Authors:** Camillo Ricordi, Francesca Pacifici, Giacomo Lanzoni, Anna Teresa Palamara, Enrico Garaci, David Della-Morte

**Affiliations:** 1 Diabetes Research Institute, Cell Transplant Center, University of Miami Miller School of Medicine, Miami, FL 33136, USA; ricordi@miami.edu (C.R.); glanzoni@med.miami.edu (G.L.); 2 Department of Systems Medicine, University of Rome “Tor Vergata”, 00133 Rome, Italy; francesca.pacifici@uniroma2.it; 3 Department of Biochemistry and Molecular Biology, University of Miami Miller School of Medicine, Miami, FL 33136, USA; 4 IRCCS San Raffaele Pisana, 00166 Rome, Italy; annateresa.palamara@uniroma1.it; 5 Department of Public Health and Infectious Diseases, Sapienza University of Rome, Laboratory Affiliated to Institute Pasteur Italia-Cenci Bolognetti Foundation, 00185 Rome, Italy; 6 San Raffaele Roma Open University, 00166 Rome, Italy; enrico.garaci@sanraffaele.it; 7 Department of Neurology and Evelyn F. McKnight Brain Institute, Miller School of Medicine, University of Miami, Miami, FL 33136, USA

**Keywords:** COVID-19, metabolic diseases, natural supplements

## Abstract

COVID-19 is without any doubt the worst pandemic we have faced since the H1N1 virus outbreak. Even if vaccination against SARS-CoV-2 infection is becoming increasingly available, a more feasible approach for COVID-19 prevention and therapy is still needed. Evidence of a pathological link between metabolic diseases and severe forms of COVID-19 has stimulated critical reflection and new considerations. In particular, an abnormal immune response observed in certain patients with SARS-CoV-2 infection suggested possible common predisposing risk factors with autoimmune diseases such as Type 1 Diabetes (T1D). Correct supplementation with dietary factors may be key to preventing and counteracting both the underlying metabolic impairment and the complications of COVID-19. A set of agents may inhibit the cytokine storm and hypercoagulability that characterize severe COVID-19 infection: vitamin D3, omega-3 polyunsaturated fatty acids, polyphenols like pterostilbene, polydatin and honokiol, which can activate anti-inflammatory and antioxidant sirtuins pathways, quercetin, vitamin C, zinc, melatonin, lactoferrin and glutathione. These agents could be highly beneficial for subjects who have altered immune responses. In this review, we discuss the antiviral and metabolic effects of these dietary factors and propose their combination for potential applications in the prevention and treatment of COVID-19. Rigorous studies will be fundamental for validating preventive and therapeutic protocols that could be of assistance to mitigate disease progression following SARS-CoV-2 infection.

## 1. Introduction: COVID-19, Metabolic Disease and Diabetes

In January 2020, a novel betacoronavirus known as Severe Acute Respiratory Syndrome Coronavirus 2 (SARS-CoV-2) was identified in China and found to be the pathogenic agent responsible for a pneumonia-like disease termed Coronavirus Disease 19 (COVID-19) [1]. COVID-19 spread rapidly and dramatically worldwide, causing 117 million confirmed cases at the time of this writing [2]. 

SARS-CoV-2 is a single-strand RNA virus encoding for 4 main structural proteins: spike (S), envelope (E), membrane (M), and nucleocapsid (N) [3]. The S-protein is composed of subunits S1 and S2 and plays a relevant role in viral infection by mediating both attachment and fusion between the viral particle and host cell [4]. The S-protein is proteolytically cleaved by host proteases (such as furin, trypsin, cathepsin) at the S1/S2 site [4], which promotes the binding of the S1 subunit to an angiotensin converting enzyme 2 (ACE2) receptor [4]. Following infection, individuals with poorly balanced immune responses experience an overproduction of pro-inflammatory cytokines (tumor necrosis factor [TNF] alpha, IL-6, and IL-1β), described as a cytokine storm, which leads to an increased risk of vascular hyperpermeability, multiorgan failure, and eventually death [4].

**Figure 1:**
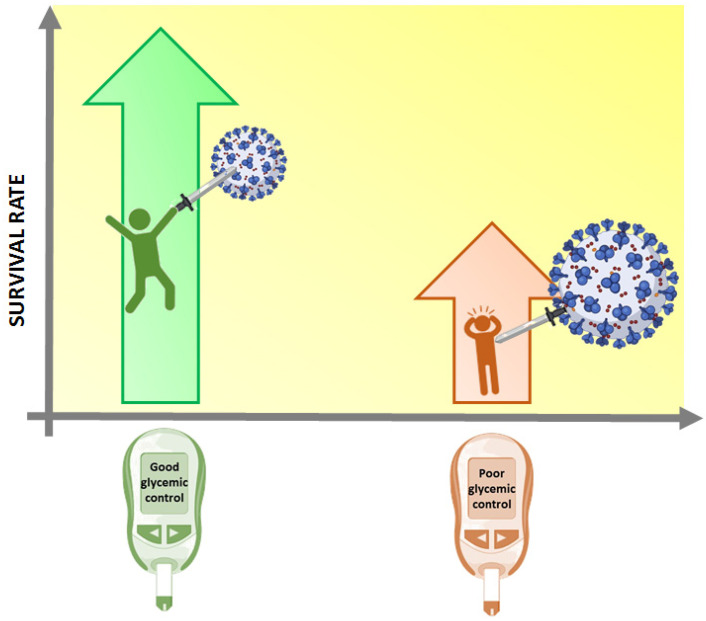
Impact of COVID-19 on patients with diabetes. Survival rate is dramatically reduced in subjects with diabetes infected with SARS-CoV-2. Created with BioRender.com.

Besides the lungs, ACE2 receptors are widely expressed in other organs, including the pancreas (beta cells), adipose tissue, liver, and kidneys, all of which are highly related to metabolism, thus suggesting a relationship between COVID-19 and metabolic diseases, such as diabetes [4,5]. However, even if mortality is dramatically increased in patients with metabolic diseases [6] (Figure 1), studies analyzing the direct effect of SARS-CoV-2 on beta cell function and the loss of metabolic homeostasis are scarce. Recently, a single center trial in COVID-19 patients has been proposed by the University of Milan, Italy, with the goal of testing whether SARS-CoV-2 infection and COVID-19 are deleterious to beta cell function [7]. Important data is expected from this trial. 

The vast majority of clinical studies demonstrated the tremendous impact of COVID-19 in patients with diabetes. Although most clinical studies in the field of COVID-19 and diabetes have focused on type 2 diabetes, emerging data suggest that type 1 diabetes (T1D) [8] may interact with SARS-CoV-2 and lead to autoimmune diseases due to the autoantibody-mediated destruction of beta cells [9]. In a multicenter surveillance study conducted in the U.S., diabetic ketoacidosis was shown to be the most relevant adverse outcome in subjects infected with SARS-CoV-2 compared to the non-infected group [10]. 

Moreover, in 2017, the TEDDY (The Environmental Determinants of Diabetes in the Young) study already reported subjects with respiratory infection, such as those caused by seasonal influenza or a coronavirus, showed an increased risk of beta cell autoimmunity, suggesting a possible bidirectional relationship between metabolic diseases and the COVID-19 pandemic [11]. Accordingly, it has been recently reported that both human pluripotent stem cell (hPSC)-derived endocrine cells and primary adult human islets allow SARS-CoV-2 entry [12]. Furthermore, in hPSC-derived endocrine cells a significant increase in chemokine expression similar that observed in autopsies of COVID-19 patients has been showed [12]. 

A reduction in protective factors associated with the evolution of unhealthy western diets could represent a shared physiopathological background that may link diabetes to COVID-19. Restoration of these factors through healthier diets could help develop preventive strategies by reducing risk factors that could affect disease progression or severity, in autoimmune (e.g., T1D), viral (e.g., coronavirus-induced) and age-related chronic diseases. 

Since we are facing a pandemic caused by a highly contagious virus, and since vaccination has been only recently available the long-term effects of which still need to be defined, there is a critical need to improve our knowledge of how selected nutrients and protective substances could affect COVID-19 susceptibility, progression, and outcome.

## 2. Diet and Protective Substances

Recently, the World Health Organization (WHO) suggested how appropriate nutrition and selected protective substances could modulate and improve the ability of the immune system both to mitigate disease progression and severity and to recover from viral infections, such as COVID-19 [13]. A recent study suggested that dietary differences among European countries could affect COVID-19-related mortality. Specifically, countries with a higher consumption of antioxidants or food products with anti-ACE activity (such as cabbage or fermented milk) showed lower mortality rates compared to countries with a lower consumption [14]. Therefore, an inappropriate diet may predispose a person to hyperinflammatory and hyperimmune reactions, thereby increasing the risk of severe disease progression in COVID-19. Based on these findings, we are promoting the “Fit4Pandemic” initiative (https://fit4pandemic.com (accessed on 15 March 2021)), to help strengthen our immune defenses, while avoiding hyperimmune and hyperinflammatory responses. The selected protective factors include vitamin D, vitamin C (liposomal), omega-3 fatty acids (eicosapentaenoic acid (EPA) and docosahexaenoic acid (DHA)), zinc and polyphenols (polydatin and pterostilbene) combined with a healthy Mediterranean, anti-inflammatory diet, and physical exercise programs to improve fitness.

Although randomized controlled trials (RCTs) will be needed to validate the proposed recommendations, the molecules under consideration have already been shown to modulate inflammation and prevent hypercoagulation. In addition, they have demonstrated antioxidant, anti-aging and antimicrobial properties, all of which address typical complications of COVID-19. Front-line health care workers and several subjects at risk of infection are following the abovementioned, common sense suggestions to keep their immune systems “fit”, but under no circumstances can they be considered therapeutic interventions.

### 2.1. Vitamin D3

Several beneficial effects of vitamin D3 have been demonstrated beyond its well-established role in the maintenance of bone homeostasis [15]. A relevant role has been found in patients with T1D [15,16] because significantly lower levels of vitamin D3 were observed in T1D subjects compared to controls [16]. In pre-clinical models of T1D, vitamin D3 supplementation improved fasting glycemia, insulin secretion, and suppressed beta cell apoptosis [17,18]. Moreover, vitamin D3 supplementation blunted inflammation and reduced T cell recruitment, leading to a decrease in the autoimmune process [16], suggesting a possible role in immune- and inflammatory-mediated diseases. In line with this finding, vitamin D3 administration reduced the activation of Th1 cells (especially Th17, which plays a pivotal role in T1D autoimmune response) [19], leading to a reduction in beta cell death [16] (Table 1).

Reduced levels of vitamin D3 have been also associated with respiratory infections and more adverse outcomes were linked to the enhancement of cytokines storms induced by hypovitaminosis D [20]. Therefore, a link between low vitamin D3 levels and COVID-19 has been proposed [21]. Furthermore, hypovitaminosis D has been significantly correlated with increased severity of COVID-19 outcomes [22]. Moreover, low levels of vitamin D3 were associated with anosmia and ageusia [23], which have been reported as early features of SARS-CoV-2 infection [24]. Although RCTs are need to deeply investigate the mechanism linking COVID-19 pandemic with hypovitaminosis D, the beneficial effect of vitamin D3 supplementation on respiratory infection has already been reported [25], suggesting that vitamin D3 prophylaxis may be helpful against coronavirus infection. 

### 2.2. Omega-3 Fatty Acids (EPA and DHA)

Chronic supplementation with high doses of omega-3 polyunsaturated fatty acids (PUFAs) have been associated with both improved glycemic control and insulin sensitivity and with a reduced risk of developing diabetes [26]. Interestingly, the DAYSY (Diabetes Autoimmunity Study in the Young) study demonstrated that PUFA supplementation starting at age 1 significantly reduced the risk of islet autoimmunity in relatives of subjects with T1D [27] (Table 2). The explanation of the immunomodulatory effect of PUFAs observed in T1D has been recently deeply analyzed in a pre-clinical model of T1D showing that dietary supplementation PUFAs (docosahexaenoic acid (DHA) and eicosapentaenoic acid (EPA) reversed autoimmune diabetes. In human subjects, peripheral blood mononuclear cells exposed to DHA and EPA showed an increase in regulatory T cell reduction in Th1, particularly Th17 cells, and an increase in Th2 activation [28]. 

The anti-inflammatory and immunomodulatory effects of PUFAs could affect COVID-19 progression and disease severity [29]. In fact, it has been reported that high doses of both DHA and EPA may decrease both IL-6 and IL-1β [30], proteins that play a central role in cytokine storms induced by COVID-19 [31]. Moreover, pre-clinical models of acute respiratory distress syndrome (ARDS), a well-known complication of COVID-19 [32], demonstrated that an omega-3 enriched diet resulted in reduced pulmonary neutrophil infiltration and lower pulmonary permeability [33] (Table 2). Oral administration of aspirin has been suggested for improving the anti-inflammatory effect of omega-3 PUFAs and increasing the release of resolvins [34], the omega-3 PUFAs active metabolites that promote resolution of the acute inflammatory phase [35,36]. However, clinical studies, especially RCTs that report on the beneficial effect of omega-3 PUFAs on COVID-19, are still missing. To the best of our knowledge, there has been only one clinical trial that evaluated the effect of EPA (on 240 hospitalized SARS-CoV-2-positive subjects) [37], but the results have not yet been reported. The main outcomes analyzed in this trial are the respiratory functions (such as oxygen saturation), inflammatory state (with a focus on IL-6 levels), and mortality rates [37]. An additional study in 100 patients affected by COVID-19 reported lower mortality in those treated with high doses of DHA and EPA [38].

The well-described modulation of inflammatory mechanisms by omega-3 PUFAs at different levels, including cellular membrane and hormonal synthesis [36], could further support their role in preventing disease progression in viral infections like COVID-19 (Table 2).

### 2.3. Pterostilbene, Polydatin and Honokiol

Pterostilbene, polydatin and honokiol are polyphenols that have been widely used for their beneficial anti-inflammatory and anti-oxidant properties [39]. These agents have been used for centuries, especially in eastern medicine. Pterostilbene and polydatin are manly precursors of resveratrol, the polyphenol known for its powerful antioxidant, cardioprotective, and neuroprotective effects (as well as the so-called “French paradox”, the paradoxical observation of lower coronary artery diseases rate in French people despite high increase in saturated fatty acid consumption, probably due to the enhanced red wine intake, which contain resveratrol) [40]. Like resveratrol, these agents activate sirtuins and have been studied extensively in the field of anti-aging [41,42,43]. Recent surprising findings suggest that these agents have the potential to enhance insulin secretion and protect against viral infections. 

An analysis of agents in a library of over 200,000 natural compounds identified pterostilbene as one of the most potent inhibitors of the ability of the SARS-CoV-2 S protein to bind to ACE2 receptors [44]. Polydatin was found to bind directly to the main protease (Mpro) of SARS-CoV-2, thus inhibiting viral entry into cells [45]. 

Pterostilbene and polydatin can also have beneficial effects on metabolic diseases. In a pre-clinical model of Type 2 Diabetes (T2D), pterostilbene was found to ameliorate glycemic control, dyslipidemia, and liver injury [46]. Other studies confirmed this protective effect of pterostilbene in both in vitro and in vivo [47]. Similarly, oral administration of 50 mg/kg of polydatin in diabetic rats significantly enhanced glucose tolerance and insulin secretion [48]. Polydatin treatment enabled the preservation of cell viability, the reduction of ROS (Reactive Oxygen Species) accumulation and the enhancement of anti-oxidant, anti-apoptotic, and functional cell markers. It ultimately led to the inhibition of oxidative damage and β-cell function [48].

Honokiol has been identified as the most powerful natural compound with antithrombotic activity [49]. Hypercoagulability is one of the most important complications of COVID-19, because it can lead to thromboembolism and damage to several organs. Honokiol may be of significant value whether used for prevention or treatment in subjects affected by SARS-CoV-2. Interestingly, honokiol induced a renoprotective effect in BTBR ob/ob mice with T2D by activating SIRT3 [50]. 

Just following this evidence, it was possible to discover a multitude of molecular pathways, including viral and metabolic-related pathways. Since all these polyphenols activate sirtuins––a family of NAD-histone deacetylase composed of 7 proteins [51]––it is also possible that sirtuin activation represents a central pathway leading to beneficial effects. The combined action of these agents could have synergistic effects on sirtuin activation. The synthesis and activity of sirtuin proteins decrease with age, and this decrease leads to a series of inefficiencies in the human body that contribute to the progressive loss of personal autonomy [52]. Sirtuins can be activated by caloric restriction and physical activity, which doubled the lifespan in preclinical model systems [53]. However, sirtuins can also be activated by plant-derived natural compounds, such as pterostilbene, polydatin, and honokiol, resulting in a similar increase in lifespan [54]. The effect of sirtuins on longevity is mediated by the regulation of metabolic homeostasis at the cellular level with consequent reduction in oxidative stress and inflammation [55]. On the one hand, these observations highlight an underappreciated role of sirtuins in the pathophysiology of COVID-19; on the other hand, sirtuins may be modulated through activators for the treatment of COVID-19 as was recently proposed [56]. 

### 2.4. Quercetin, Vitamin C, Zinc, Melatonin

SARS-CoV-2 infection has been reported to induce the over-activation of Nlrp3 inflammasome, leading to cytokine storms responsible for tissue damage and severe complications from COVID-19 [57]. The Nlrp3 inflammasome is a multimeric protein complex with a central role in the activation of the innate immune system against pathogens [58]. Nlrp3 activation is also associated with several metabolic disorders, such as T2D in addition to chronic neurological diseases like Alzheimer’s and Parkinson’s [45,58]. Recently, natural compounds were reported to have reduced cytokine release by controlling Nlrp3 activation [59]. 

**Figure 2:**
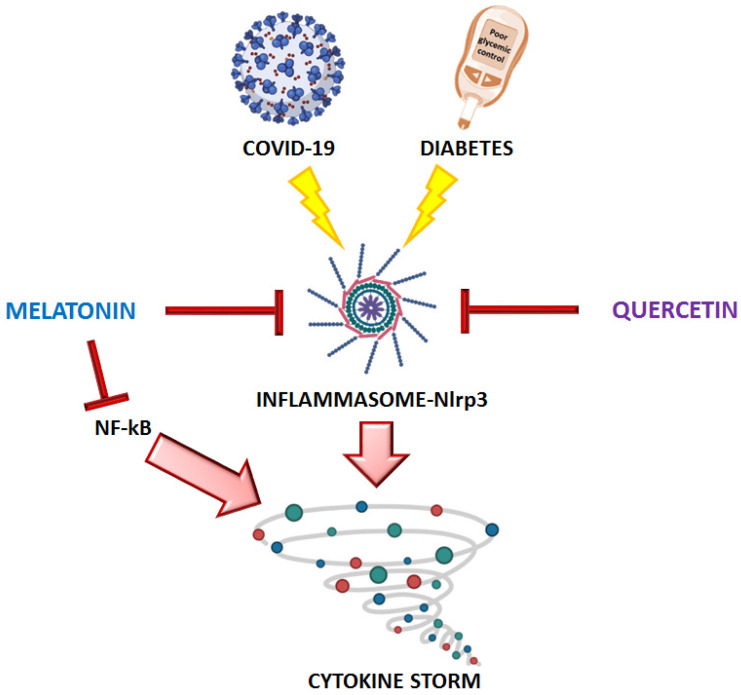
Proposed protective effects for melatonin and quercetin against SARS-CoV-2 infection. Melatonin and quercetin may blunt the deleterious effects of SARS-CoV-2 by reducing activation of the cytokine storm. Created with BioRender.com.

Among these, quercetin, a flavonoid found in several vegetables and fruit, produced an important anti-inflammatory and anti-oxidative effect [59]. In animal models, quercetin administration led to a reduction in Nlrp3 activation and related inflammatory processes, leading to improvements in several pathological conditions [59]. Interestingly, quercetin also showed robust anti-viral activity against respiratory and several others virus, by acting at different levels: virus entry, virus assembly and protease activity [60]. According to these findings, quercetin has been proposed as a possible therapeutic approach against SARS-CoV-2 infection [60] (Figure 2).

Melatonin is a hormone molecule produced mainly during the night with the highest release in the mid-dark period [61]. Melatonin has been shown to have an anti-inflammatory effect by activating molecules such as Sirtuin 1 and by reducing the activation of pro-inflammatory factor NF–kB, which promotes the release of several inflammatory cytokines [61]. In a rat model of ventilator-induced lung injury, melatonin receptor–agonist administration blunted NF–kB activation and led to a decrease in TNFα, IL-1β, and IL-6 secretion [62]. Since these inflammatory cytokines are released in high amounts following SARS-CoV-2 infection, it is plausible that melatonin could have a protective role against a severe COVID-19 infection. Moreover, melatonin was found to boost the immune system by reducing Nlrp3 activation and by promoting proliferation and maturation of immune cells, including natural killer cells, granulocytes, and monocytes [63] (Figure 2). Melatonin production decreases with age; hence, the benefits of melatonin against SARS-CoV-2 infection may be maximized in the elderly population, which is the most affected by severe COVID-19 complications. 

Vitamin C, also known as ascorbic acid, is an essential nutrient with relevant anti-viral properties [60]. In a murine model of influenza virus infection, vitamin C administration reduced both mortality and lung pathology [60]. A beneficial effect of ascorbic acid has also been reported in virus-related acute respiratory distress syndromes [60]. Based on these observations, a clinical trial has been proposed to analyze the effect of vitamin C supplementation in reducing the in-hospital mortality of patients with pneumonia induced by SARS-CoV-2 infection [64]. Moreover, quercetin and vitamin C could be combined to yield a synergistic effect against COVID-19 [60]. The use of vitamin C as a preventive supplement against COVID-19 has a strong rationale, as we previously reported [65].

Zinc, an essential micronutrient found in several kinds of food, has a well-established role in immune system functions [66]. Because zinc depletion boosts pro-inflammatory processes that cause tissue damage [67], low zinc levels are associated with acute respiratory failure in the elderly and with acute lower respiratory tract infection in children [68,69]. Moreover, zinc depletion has been observed in disorders that are the main comorbidities observed in COVID-19 patients [66]. Zinc supplements could thus be beneficial for preventing COVID-19 pathogenesis or inhibiting its progression to a severe disease, as previously suggested [70].

### 2.5. Lactoferrin and Glutathion

Lactoferrin is a protein mainly found in milk, in several exocrine secretions (such as saliva), and in certain cells types, including neutrophils [71]. Lactoferrin showed relevant anti-microbial activity and an ability to enhance immune defense systems against several pathogens, including viruses [72]. During autoimmune pancreatitis, antibodies targeting lactoferrin are produced [73], which suggests that autoimmunity plays a role in attacking the pancreas. Autoantibodies against lactoferrin are also observed frequently in T1D [74], suggesting the possible involvement of lactoferrin deficiency in the development of T1D [74]. Due to its immunomodulatory and anti-inflammatory properties, lactoferrin has also been suggested for use in therapeutic approaches against COVID-19. Liposomal lactoferrin was studied, because liposomes enhance lactoferrin function by reducing its clearance [75]. In a study conducted on 75 non-hospitalized symptomatic COVID-19-positive patients, liposomal lactoferrin supplements yielded improvements in recovery of parameters such as tiredness, muscular pain, taste, and smell [76] (Table 3).

Glutathione is an antioxidant that counteracts oxidative stress and improves immune cell function [77]. It has been reported that glutathione levels are dramatically reduced in young subjects with poorly controlled T1D because it is used up at an increased rate [78]. More recently, a study showed that not only total glutathione, but also its other forms (i.e., reduced, oxidized, and protein-bound) were reduced in subjects with T1D [79]. These findings suggested that glutathione depletion increases oxidative damage from diabetes; moreover, the alteration of glutathionylated proteins and related pathways may contribute to diabetes-related complications [79]. Besides its antioxidant activity, which enhances the function of several cells of the immune system, glutathione also shows anti-viral activity that leads to a reduced viral load [80]. Several factors may contribute to glutathione depletion, including age (glutathione levels decrease significantly with age), sex (reduced active glutathione is lower in men than in women), and chronic conditions (e.g., obesity, diabetes, and cardiovascular disease) [80]. Notably, these factors are also the main risk factors for severe COVID-19 and are associated with a poor prognosis [81,82,83]. In a study conducted on mild to severe COVID-19 patients, glutathione levels were significantly reduced in those with the most severe symptoms compared to the mild symptomatic patients [80]. Moreover, an increase in the ROS and in ROS/glutathione ratio was observed [80]. These preliminary findings suggest that glutathione depletion could have a substantial impact on COVID-19 progression (Table 4).

### 2.6. Curcumin and Piperine

Curcumin is a polyphenol with well-established anti-oxidant and anti-inflammatory activities [84]. Due to these properties, the effect of curcumin administration has been reported in diabetes. In particular, in a mice model for T1D induced by streptozotocin administration, curcumin pre-treatment significantly blunted pro-inflammatory cytokines sera levels and maintained the euglycemic state [85]. Moreover, in isolated pancreatic islets treated with pro-inflammatory cytokines, curcumin pre-treatment increased islet viability [85]. Based on its anti-inflammatory activity, several studies analyzed the potential involvement of curcumin against COVID-19. A computational study conducted by Jena and colleagues, demonstrated that curcumin may interact with the spike protein of SARS-CoV-2 virus potentially preventing viral infection [86]. Moreover, curcumin was also found to interfere with the activity of several proteases for the cleavage of the spike protein, leading to a reduction in viral entry [87]. Interestingly, curcumin treatment leads to a significant decrease of the influenza virus A-induced release of IL-6 and TNF-α. These two inflammatory mediators are key activators of the COVID-19 cytokine storm; hence, curcumin may be beneficial for inhibiting it [88]. Curcumin supplementation has been proposed to improve outcomes in COVID-19 [89]. 

Piperine is an active polyphenolic compound, found in the black pepper, which exerts a potent anti-inflammatory effect. It has been shown that piperine reduced significantly the release of pro-inflammatory cytokines such as IL-6 in a model of rheumatoid arthritis [90]. Based on these findings, a randomized blind clinical trial was proposed to assess the impact of the curcumin/piperine co-supplementation on the main outcomes of COVID-19. The trial aims to boost the anti-inflammatory activity of these phenolic compounds in order to counteract the clinical symptoms and the severity of the SARS-CoV-2 infection [89].

## 3. Conclusions

COVID-19 is a complex infectious disease that can lead to severe multiorgan impairment and death. Age and comorbidities are the most important negative correlates associated with the worst outcomes of COVID-19, including a high mortality rate. Among comorbidities, metabolic diseases such as diabetes play a tremendous role by boosting the inflammatory and hypercoagulative cascade. This is particularly evident in patients with a long history of diabetes and poorly controlled T1D. The severe outcomes observed in patients with T1D and T2D may be connected to an elevated basal inflammatory state, and to aberrant immune responses after SARS-CoV-2 infection. 

One of the most promising strategies for prevention and treatment is based on controlling inflammation, oxidative stress, and immune responses via specific nutrients and natural compound supplements, which are beneficial either for the underlying diseases or for COVID-19 itself. A combination of supplements and nutrients that constitute a unique compound able to act at different levels of COVID-19 physiopathology, is currently under investigation. The goal of such a combination is to stimulate activation of molecular pathways: sirtuins reduce inflammation and oxidative stress; pterostilbene, polydatin and honokiol reduce the prothrombotic effect imparted by the virus; and zinc and omega-3 PUFAs modulate the immune response and inflammatory cytokine storm. Preliminary results obtained from a variety of model systems as well as from COVID-19 patients appear extremely promising. From nature sometimes comes danger, but from nature also comes the means to fend it off. 

## Figures and Tables

**Table 1:** Evidence related to low levels of vitamin D in T1D, respiratory infection and its putative association with COVID-19.

VITAMIN D3		
T1D	Respiratory Infection	Possible Role in COVID-19
↓ levels of vitamin D3 vitamin D3 supplementation: ✓ ↓ fasting glycemia ✓ ↑ insulin secretion ✓ ↓ autoimmune process	↓ levels of vitamin D3 ↑ adverse outcomes ↑ cytokine storm	↓ levels of vitamin D correlated with COVID-19 severity ↓ levels of vitamin D are associated with anosmia and ageusia

↑: increased; ↓ reduced.

**Table 2:** Evidences related to PUFA supplementation in T1D, respiratory infection and its possible association with COVID-19.

OMEGA-3 FATTY ACIDS (EPA + DHA)		
T1D	Respiratory Infection	Possible Role in COVID-19
PUFA supplementation: ✓ ↓ inflammatory response ✓ ↓ autoimmunity	PUFA supplementation: ✓ ↓ pulmonary neutrophils infiltration ✓ ↓ pulmonary permeability	↓ COVID-19 related ARDS ↓ COVID-19-induced cytokine storm

ARDS: acute respiratory distress syndrome; ↑: increased; ↓ reduced.

**Table 3:** Evidences related to lactoferrin’s role in T1D, and its possible association with COVID-19.

LACTOFERRIN		
General Function	T1D	Possible Role in COVID-19?
✓ anti-microbial activity ✓ enhance immune defenses systems	↑ antibodies against lactoferrin	Lactoferrin supplementation ↑recovery of patients (tiredness, muscular pain, taste and smell)

↑: increased; ↓ reduced.

**Table 4:** Evidences related to glutathione’s role in T1D, and its possible association with COVID-19.

GLUTATHIONE		
General Functions	T1D	Possible Role in COVID-19
✓ enhances immune cell function ✓ antiviral activity	↓ glutathione levels ↑ oxidative damage	↓ glutathione levels ↑ viral load

↑: increased; ↓ reduced.
